# Identification and characterization of the gene *OvANS* associated with purple flower in *Orychophragmus violaceus*

**DOI:** 10.1186/s12870-025-07412-x

**Published:** 2025-10-09

**Authors:** Yi Liu, Chenchen Wang, Haidong Chen, Wenjie Shen, Lunlin Chen, Chen Tan, Daozong Chen

**Affiliations:** 1 College of Life Sciences, Ganzhou Key Laboratory of Greenhouse Vegetable, Gannan Normal University, Ganzhou, 341000 China; 2 Nanchang Branch of National Center of Oilcrops Improvement, Province Key Laboratory of Oil Crops Biology, Crops Research Institute of Jiangxi Academy of Agricultural Sciences, Nanchang, 330200 Jiangxi China

**Keywords:** Orychophragmus violaceus, Flower color, RNA-seq, Anthocyanin biosynthesis, OvANS, Insertions in the promoter region

## Abstract

The color of flowers plays a crucial role in ornamental plant breeding, impacting its market value significantly. *Orychophragmus violaceus*, a traditional ornamental flower cultivated for its early blooming in spring, often exhibits white flower variations during the breeding process, enhancing its ornamental appeal. This study systematically identified 98 genes associated with anthocyanin biosynthesis in *O. violaceus*, distributed across 12 chromosomes. Secondly, we conducted an analysis of expression patterns and differentially expressed genes (DEGs) of anthocyanin biosynthetic pathway-related genes in *O. violaceus* by utilizing comparative transcriptome data from white (OvW-1) and purple (OvP-37) flower petals. Our findings revealed significant differential expression of genes *ANS*, *GST*, *TCP3* and *AHA10* between the white and purple flower colors. Subsequently, we employed qRT-PCR to validate the differential expression of *OvANS* and *OvTCP3* in OvW-1 and OvP-37 flowers, with *OvANS* showing significantly higher expression levels in OvP-37 plants. Moreover, we successfully cloned the complete sequence of the *OvANS* gene from OvW-1 and OvP-37 flowers, revealing the presence of two inserted fragments in the promoter region of the *OvANS* gene in white flowers, potentially playing a crucial role in gene expression repression. Subsequently, we conducted qRT-PCR analysis to investigate the expression pattern of the *OvANS* gene in various tissues (leaves, petioles, petals, and young siliques) of OvW-1 and OvP-37 flowers. Our findings demonstrated that *OvANS* was ubiquitously expressed in all four tissues of OvP-37, with the highest expression level observed in petals, whereas its expression in OvW-1 was negligible. Our results provide new insights into the molecular mechanism of flower color variation in *O. violaceus*, and provide a reference for enriching the flower color types of *O. violaceus*.

**Supplementary Information:**

The online version contains supplementary material available at 10.1186/s12870-025-07412-x.

## Introduction

Flavonoids, including anthocyanins, constitute a diverse group of metabolites found in plants, serving as the predominant pigments responsible for the orange, red, purple, pink, and blue hues observed in various tissues of flowering plants across different species. Anthocyanins, in particular, are recognized for their pivotal contributions to plant defense mechanisms and human health. These compounds are integral to a range of biological responses to both biotic and abiotic stressors, including disease resistance, UV-B protection, and mitigation of herbivory by insects and mammals [1]. Anthocyanins play a crucial role in attracting insects and animals for pollination and seed dispersal through the resulting colors in flowers [2]. Additionally, they are increasingly recognized for their significance as quality traits in ornamental plants and fruit crops, not only for their aesthetic value but also for their potential health benefits to humans. Studies have shown that anthocyanins may contribute to a reduced risk of various health issues, including obesity-related diseases, cancers, diabetes, and degenerative conditions like Alzheimer’s disease [3–5].

Anthocyanin biosynthesis is a complex process that is initiated through the flavonoid synthetic pathway, involving the activation of multiple enzymes encoded by biosynthetic genes. This pathway can be categorized into three distinct phases: the initial steps of the general phenylpropanoid pathway, the subsequent early steps of the flavonoid pathway, and the final late steps of the anthocyanin-specific pathway [6]. The initial steps of the phenylpropanoid pathway involve a series of three enzymatic reactions converting phenylalanine to 4-coumaroyl CoA via cinnamic acid and coumaric acid, facilitated by the enzymes phenylalanine ammonia-lyase (PAL), cinnamate-4-hydroxylase (C4H), and 4-coumaroyl CoA: ligase (4CL). Subsequently, the early flavonoid biosynthetic reactions are mediated by early biosynthetic genes (EBGs), including chalcone synthase (CHS), chalcone isomerase (CHI), and flavonol 3-hydroxylase (F3H). Subsequently, Shi and Xie (2014) identified late biosynthetic genes (LBGs) responsible for encoding dihydroflavonol-4-reductase (DFR), anthocyanidin synthase (ANS; also called leucoanthocyanidin dioxygenase, LDOX), and UDP-glucose: flavonoid-3-O-glycosyl-transferase (UF3GT). Co-regulation of pathway genes involved in flavonoid biosynthesis was observed [7–10]. The initial steps of the general phenylpropanoid pathway were found to be negatively regulated by the R2R3-MYB subgroup 4 (*MYB3*, *MYB4*, *MYB7* and *MYB32*) [11, 12], while the early biosynthetic genes (EBGs) were regulated by three redundant R2R3-MYB transcription factors (TFs) *MYB11*, *MYB12*, and *MYB111* [9, 13]. The regulation of LBGs is governed by the ternary MYB-bHLH-WD40 (MBW) protein complex, consisting of R2R3-MYB, basic helix-loop-helix (bHLH), and WD40-repeat proteins [4, 14, 15]. In Arabidopsis, the confirmed positive transcription factors comprise production of *anthocyanin pigmentation 1* (*PAP1*)/*MYB75*, *PAP2*/*MYB90*, *MYB113*, *MYB114*, *transparent testa 2* (*TT2*), while the negative transcription factors include *caprice* (*CPC*) and *MYB-like 2* (*MYBL2*) [15–17]. The bHLH transcription factors encompass *TT8*, *glabra 3* (*GL3*), and *enhancer of glabra 3* (*EGL3*), with the WD40-repeat protein transparent testa glabra 1 (TTG1) also being identified [15, 18, 19].

The *Orychophragmus violaceus* (L.) O. E. Schulz (2n = 24, OO genomes), a Brassicaceae species native to China, is classified as an annual herb and is utilized in China as both an ornamental plant and leaf vegetable. This plant is distinguished by its high oil quality, characterized by elevated levels of palmitic and oleic acids, and reduced levels of linolenic, erucic, linoleic, and dihydroxy C24 fatty acids [20]. *O. violaceus* has been identified as a potential industrial oilseed crop due to the superior high temperature lubricant properties of its oil in comparison to castor oil, a well-established vegetable oil used in bio-based lubricant applications [20]. Concurrently, *O. violaceus* is extensively grown as a decorative plant due to its late winter flower, which bears a resemblance to the purple and white varieties, characterized by its large size and extended blooming period. Commonly known as Chinese violet cress or “eryuelan” (February orchid), *O. violaceus* has been utilized in the creation of intergeneric sexual hybrids with cultivated Brassica species to study chromosome stability [21]. Through this research, a variety of Brassica aneuploids, including hypoploids and those containing alien additions or substitutions, have been identified within the hybrid offspring [22]. Recent research has revealed that the *O. violaceus* transcript *OvPAP2*, under the control of the petal-specific promoter XY355, results in the production of red anthers and red petals in transgenic *Brassica napus* plants [23]. The decoding of the genome of *O. violaceus* offers a foundation for investigating the molecular mechanisms underlying its exceptional agronomic characteristics [24, 25].

In the realm of ornamental horticulture, flower color stands out as a pivotal characteristic that significantly impacts the commercial worth of plants. Through the breeding of *O. violaceus*, we successfully developed an inbred line showcasing the wild-type purple petals (OvP-37) alongside its natural mutant counterpart with white petals (OvW-1). This study seeks to elucidate the genetic mechanisms underlying anthocyanin biosynthesis in the purple-flowered *O. violaceus*, thereby enhancing its aesthetic appeal and overall ornamental value. Initially, a genome-wide analysis was performed on the anthocyanin biosynthesis pathway genes in *O. violaceus*, encompassing both structural genes and transcriptional regulators. Subsequent to this analysis, a comparison was made between the transcriptomes of white and purple flowers of *O. violaceus*. The findings revealed significant differential expression of four anthocyanin biosynthetic pathway-related genes in the petals of white and purple *O. violaceus*, with *OvANS* showing minimal expression in the discolored petals, a result further validated through qRT-PCR analysis. Furthermore, we successfully cloned the full-length sequence of *OvANS* and identified two significant fragment insertions in the *OvANS* promoter region of *O. violaceus*, suggesting a potential role in regulating *OvANS* expression. Subsequent analysis of *OvANS* expression patterns in various tissues of OvW-1 and OvP-37 selfing lines revealed minimal expression of *OvANS* in the tissues of OvW-1. These findings contribute valuable insights towards understanding the underlying mechanisms of flower color variation in *O. violaceus*.

## Materials and methods

### Plant materials and growth conditions

Seeds of the wild-type *O. violaceus* inbred line with purple petals (OvP-37) and its natural mutant inbred line with white petals (OvW-1) were grown in the greenhouse of the Gannan Normal University in the autumn of 2022, with 16 h light/8 h dark, temperature 22 °C. The petals of flower buds waiting to open were collected from the OvP-37 and OvW-1 inbred line with three biological repeats for RNA extraction, and the petals of freshly opened were collected with three biological repeats for total anthocyanin extraction. All samples were collected and immediately frozen in liquid nitrogen.

### Total anthocyanin extraction and content analysis

All flower samples were crushed in a mixer mill (MM 400, Retsch) with a zirconia bead for 1.5 min at 30 Hz. Each 100 mg sample of powdered tissue was extracted overnight at 4℃ in 1.0 mL of an aqueous solution of 85% methanol: formic acid (Vmethanol : Vddwater : Vformic acid = 8 : 15 : 0.5). Following centrifugation at 10,000 g for 10 min, the extracts were absorbed (CNWBOND Carbon-GCB SPE Cartridge, 250 mg, 3 mL; ANPEL, Shanghai, China, www.anpel.com.cn/cnw), filtrated (SCAA-104, 0.22 μm pore size; ANPEL, Shanghai, China, http://www.anpel.com.cn/), and used for total content analysis. The detailed method is shown in Chen et al., 2022 [26].

### RNA-seq data analysis

The RNA-seq raw data were sequenced by Bioyi Biotechnology Co., Ltd. (Wuhan, China). Low-quality reads were removed with default parameters using Trimmatic (v0.39) [27]. Clean reads were aligned to the reference genome of *O.violaceus* using the HISAT2 software (v2.1.0) [28]. The obtained millions of fragments per thousand bases (FPKM) values were then calculated using StringTie (v2.1.1) [28]. The expression histogram was generated using Excel, while the heat map was generated using TBtools-II software (v2.047) [29]. The R package DEseq2 (1.16.1) [30] was used to identify the differentially expressed genes (DEGs) between flowers of different colors based on the following criteria: padj < 0.01 & log2FoldChange > 2.

### Identification and expression analysis of anthocyanin-related genes

Here, all the protein sequences and CDS sequences of *Arabidopsis thaliana* and *O. violaceus* were obtained from tair website (https://www.arabidopsis.org/) and National Genomics Data Center (https://ngdc.cncb.ac.cn/gwh). To ensure accurate identification of anthocyanin-related genes in the *O. violaceus*, initial searches were conducted using local BLASTP and local BLASTN algorithms, with a significance threshold of E < 1e − 20. Then, the screening process involved identifying candidate genes that exhibited a consistency rate exceeding 80% and a coverage rate surpassing 80%. Gene collinearity analysis was performed using MCScanX software [31]. MEGA7 software was used for evolutionary analysis [32]. TBtools-II software (v2.047) was used to draw a heat map of the FPKM values of anthocyanin-related genes [29].

### Cloning and sequence analysis of *OvANS*

To analyze the reasons for the differential expression of *OvANS* genes between OvP-37 and OvW-1 inbred lines, we used PrimerPrimer5 software to design specific primers for cloning the full-length sequence of *OvANS* in *O. violaceus*. The sequence-specific primers for cloning the promoter region are: OvANS-pF, 5′- CGAGAACCAAGAAGCATTTAT-3′; OvANS-pR, 3′- CGGAGAAGAAAACAGAGTAAG-5′. And the sequence-specific primers for cloning the promoter region are: OvANS-gF, 5′- CCACACATCATTTACTTTGCA-3′; OvANS-gR, 3′- ACTTCAGAGAAACTAAAAGCA-5′. Young leaves of OvP-37 and OvW-1 inbred lines *O. violaceus* inbred lines were selected respectively, and genomic DNA was extracted using the CTAB method. For details on amplification, single clone screening and sequencing of PCR products, see [33]. The sequences were aligned using the online tool MUSCLE (https://www.ebi.ac.uk/Tools/msa/muscle/#).

### qRT‑PCR analysis

The young leaves, petioles, siliques and petals of fresh flower buds waiting to open of OvP-37 and OvW-1 plants were collected with three biological replicates, all samples were collected and immediately frozen in liquid nitrogen for RNA extraction and qRT-PCR analysis. The primer information for qRT-PCR analysis were list in Supplementary Table 4. For detailed methods, please refer to [34] All materials of this research were collected from Gannan Normal University research base.

## Results

### Phenotypic characterization and analysis of total anthocyanin content

**Fig. 1:**
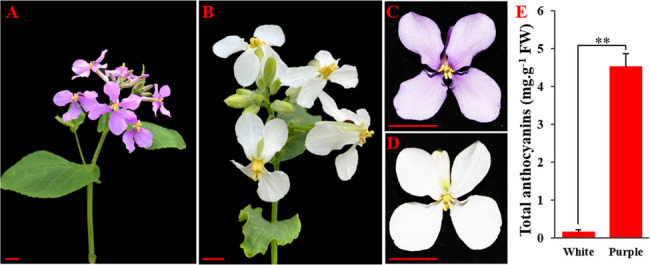
Phenotypic characterization and total anthocyanin content analysis of white and purple flower *O. violaceus*. **A**, **C**: Phenotype of purple flower *O. violaceus*; **B**, **D**༚Phenotype of white flower *O. violaceus*; **E**: Total anthocyanin content of white and purple flower *O. violaceus* petals. The scale bars in the lower right corner are 1 cm

The *O. violaceus* inbred lines OvP-37 and OvW-1 were systematically cultivated in the greenhouse at Gannan Normal University for the purpose of conducting phenotypic analysis and obtaining experimental tissues and organs. Upon the arrival of spring, *O. violaceus* initiated the process of bolting and flowering. The inflorescence of the OvP-37 inbred line displayed a profusion of purple flowers (Fig. 1A), characterized by dark purple petals (Fig. 1C). In contrast, the inflorescence of the OvW-1 inbred line showcased pure white flowers (Fig. 1B), with white petals (Fig. 1D). Subsequent to this observation, we proceeded to harvest the purple and white petals of *O. violaceus* at full bloom for the purpose of quantifying total anthocyanin content. The results showed that the total anthocyanin content in white petals was extremely low, while the total anthocyanin content in purple petals was about 4.39 mg/g fresh weight (Fig. 1E), indicating that the biosynthesis and accumulation of anthocyanin is the key to the coloring of purple flowers of *O. violaceus*.

### Genome-wide identification of genes involved in anthocyanin biosynthesis

**Fig. 2:**
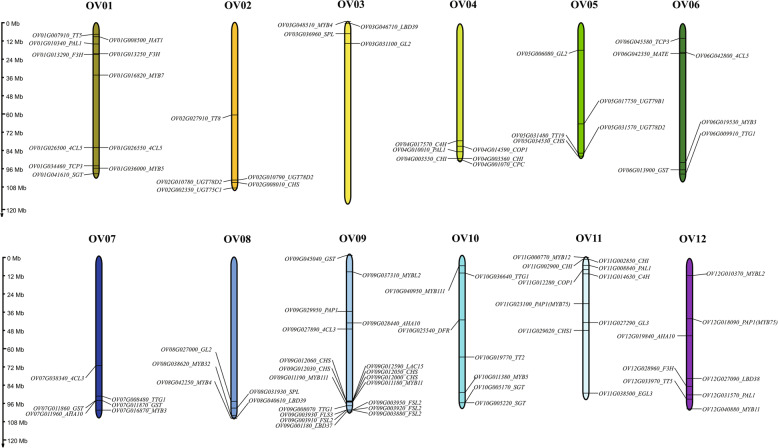
Distribution of 98 anthocyanin related genes in *O. violaceus* on 12 chromosomes

Plant anthocyanin biosynthesis pathway has been clearly reported in *A. thaliana* [35], petunia [36], *Antirrhinum majus* [37] and other species. In this investigation, a set of 52 genes associated with the anthocyanin biosynthetic pathway in *A. thaliana* were utilized as reference points to systematically ascertain the presence of analogous genes in the entire genome of *O. violaceus*. The findings revealed that 48 out of the 52 genes linked to anthocyanin biosynthesis were indeed present in the genome of *O. violaceus*, with the exceptions of *F3’H* (*AT5G07990*), *FLS1* (*AT5G08640*), *HY5* (*AT5G11260*), and *CHS3* (*AT5G17890*) which were not detected (Fig. 2; Supplementary Table 1). It is noteworthy that *O. violaceus* is a tetraploid species with homologous characteristics. Among the 48 identified anthocyanin biosynthesis-related genes, a total of 98 such genes were found to be distributed across 12 chromosomes of *O. violaceus*, indicating the presence of multiple copies of certain genes. Notably, the gene *CHS* exhibited the highest number of copies at six, while others such as *DFR*, *ANS*, *CPC*, were identified only once. Interestingly, OV09 contained 19 chromosomes, whereas OV02, OV03, and OV05 each had a distribution of only 5 chromosomes. In addition, multiple genes have tandem repeats, such as *UGT78D2*, *CHI*, *GST*, *FSL2* and *CHS* (Fig. 2).

### Analysis of the expression patterns of anthocyanin-related genes in purple and white flower *O. violaceus*

**Fig. 3:**
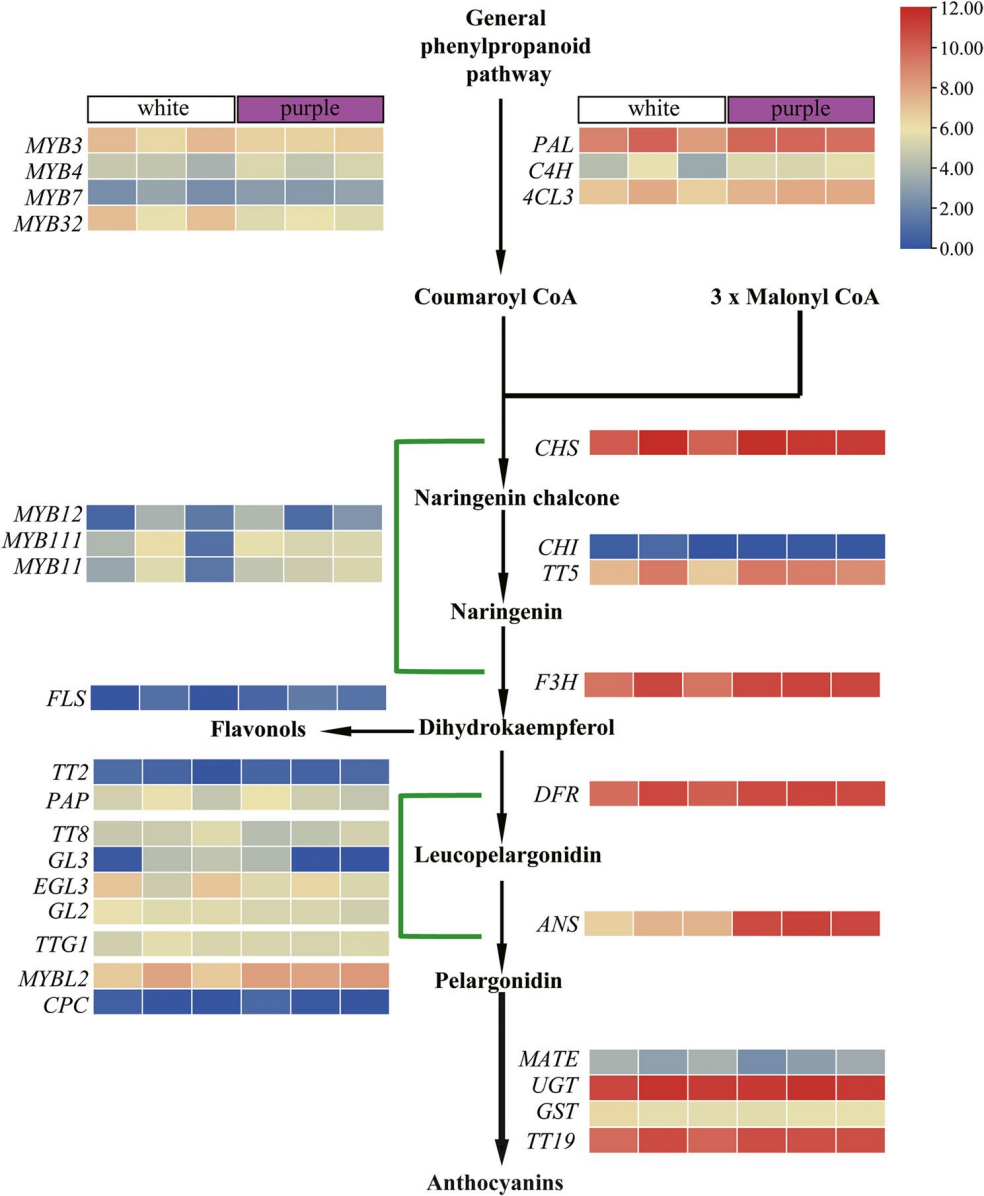
Heat map of gene expression related to anthocyanin biosynthesis pathway in *O. violaceus*

In order to investigate the expression patterns of anthocyanin genes in white and purple petals of *O. violaceus*, we conducted a comparative transcriptome sequencing analysis on the petals of OvP-37 and OvW-1. The findings revealed that the majority of genes were expressed in both white and purple petals, with a notable upregulation of structural genes involved in the anthocyanin biosynthesis pathway (Fig. 3, Supplementary Table 2). Specifically, early structural genes such as *PAL*, *C4H*, *4CL3*, *CHS*, *CHI* (*TT5*) and *F3H* exhibited higher expression levels in purple petals compared to white petals, although they were also expressed in the latter. In the latter stages of the anthocyanin biosynthesis pathway, the genes *DFR*, *ANS*, *UGT* and *GST* were observed to be expressed. Notably, the expression of *ANS* in white flowers was found to be significantly lower compared to that in purple flowers. With regard to transcription factors, *MYBL2* was found to be expressed in both white and purple petals, while the expression levels of other transcription factors were generally low and their regulatory patterns remain unclear (Fig. 3). These results suggest that although OvW-1 blooms white flowers, the anthocyanin biosynthetic pathway-related genes still have a certain expression level, resulting in a purple-to-white mutation in its flower color. It may be due to the late structural gene or transcriptional regulatory factor mutation that blocks the anthocyanin coloration modification. These findings indicate that, despite OvW-1 producing white flowers, there is still some level of expression of genes related to the anthocyanin biosynthetic pathway, leading to a mutation in flower color from purple to white.

### Comparative transcriptome analysis of purple and white flower petals of *O. violaceus*

**Fig. 4:**
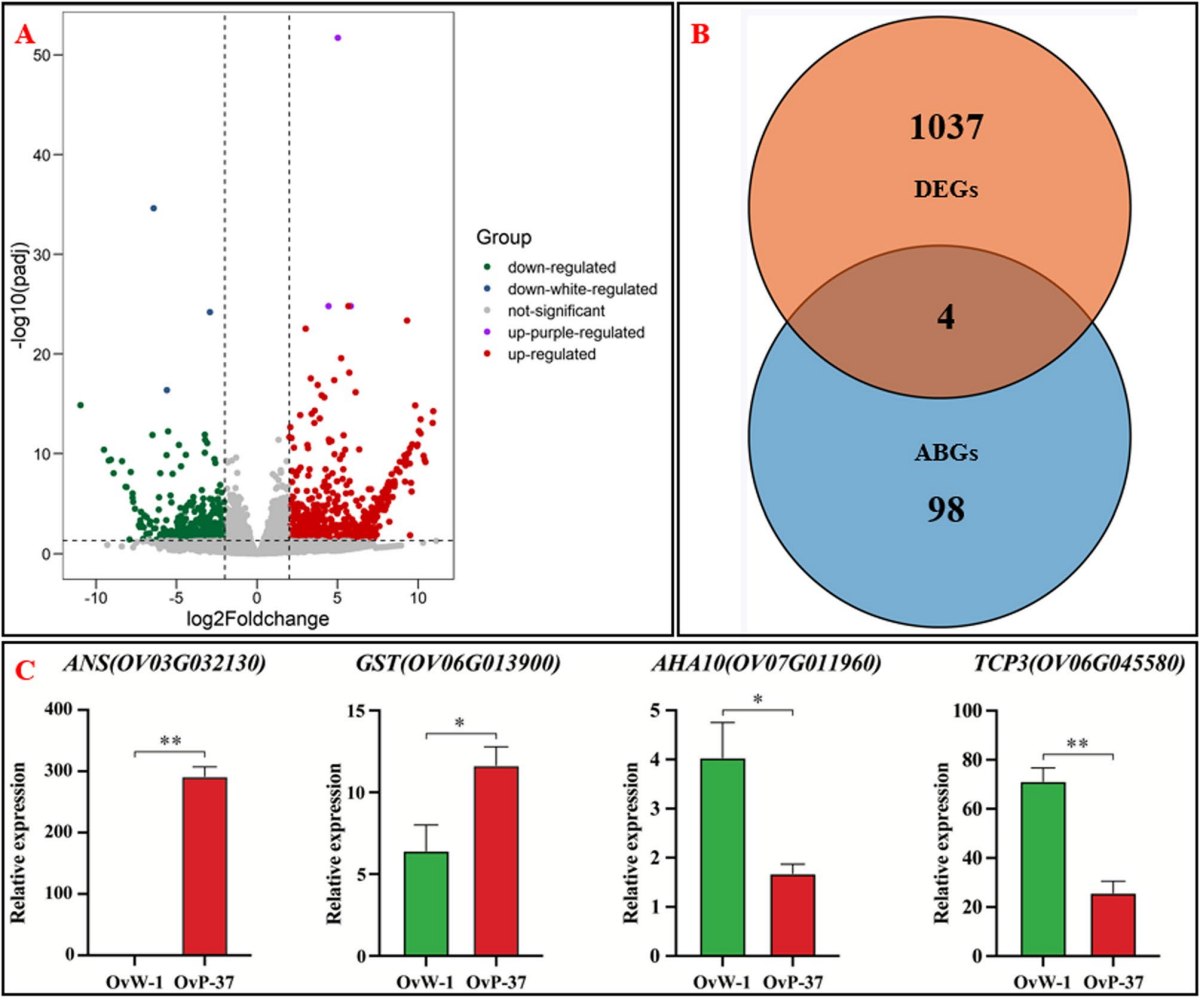
Differentially expressed genes (DEGs) analysis and quantitative verification of white and purple petals of *O. violaceus*. **A**: DEGs volcano map; **B**: The intersection Venn diagram of DEGs and anthocyanin biosynthesis related genes (ABGs) pathway; **C**: Quantitative verification by qRT-PCR

In order to further investigate the genetic basis of flower color variation in *O. violaceus*, we conducted a comparative analysis of transcriptome sequencing data from the petals of the OvP-37 and OvW-1 inbred lines. Our findings revealed differential expression of 1037 genes in the petals of white and purple *O. violaceus*, with 598 genes showing up-regulation and 439 genes showing down-regulation (Fig. 4A, Supplementary Table 3). Among the 98 genes associated with the anthocyanin synthesis pathway, 4 genes, specifically *ANS* (*LODX*), *GST*, *TCP3* and *AHA10*, exhibited differential expression in the petals of the OvP-37 and OvW-1 inbred lines (Fig. 4B; Table 1). The late structural genes *ANS* and *GST*, along with the H+-APTase AHA10 and the transcription factor *TCP3*, were examined for their expression levels using qRT-PCR. Our results show that *ANS* and *GST* exhibit high expression levels in purple petals but are either not expressed or expressed at low levels in white petals (Fig. 4C). Conversely, *AHA10* and *TCP3* are significantly up-regulated in white petals, suggesting a potential role for *ANS* and *GST* in the development of purple flowers in *O. violaceus* (Fig. 4C). Specifically, the gene *ANS* exhibited a notable up-regulation in purple petals and was found to be absent in white petals, thereby inhibiting the production of anthocyanin (Fig. 4C). This suggests that *ANS* may play a pivotal role in the genetic mutation responsible for the transition from purple to white flowers in *O. violaceus*.

**Table 1:** Anthocyanin-related differentially expressed genes

Gene ID	AT Name	Function name	Annotation
OV03G032130.2	AT4G22880	ANS	Encodes leucoanthocyanidin dioxygenase, which is involved in proanthocyanin biosynthesis.
OV06G013900.2	AT1G17170	GST	Encodes glutathione transferase belonging to the tau class of GSTs, glutathione binding and glutathione transferase activity.
OV06G045580.1	AT1G53230	TCP3	DNA-binding transcription factor activity.
OV07G011960.1	AT1G17260	AHA10	Belongs to H+-APTase gene family, involved in proanthocyanidin biosynthesis, disturbs the vacuolar biogenesis and acidification process.

### Insertion of two DNA fragments into the promoter region affects the expression of *OvANS*

**Fig. 5:**
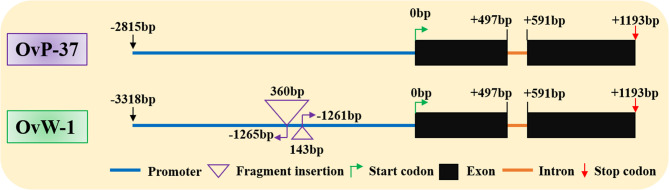
*OvANS* sequence alignment between OvP-37 and OvW-1

Prior research has demonstrated that ANS is a crucial gene involved in the biosynthesis of anthocyanins. Notably, through comparative transcriptome analysis and qRT-PCR validation of *O. violaceus* petals, it was observed that *OvANS* exhibited a significant up-regulation in purple flowers. To delve deeper into the underlying factors contributing to its differential expression, the *OvANS* genes were cloned from the OvP-37 and OvW-1 inbred lines, respectively. The findings demonstrate that *OvANS* exhibited a high degree of conservation in the OvP-37 and OvW-1 inbred lines within the gene region. Additionally, within its promoter region spanning approximately 3000 base pairs, *O. violaceus* displayed two consecutive large fragment insertions, specifically a 143 bp insertion at −1261 bp and a 360 bp insertion at −1265 bp (Fig. 5). These insertions likely contribute to the silencing of *OvANS* in *O. violaceus*, whereas the normal expression of purple flowers is attributed to the absence of sequence insertions, facilitating the normal synthesis and accumulation of anthocyanins for the presentation of purple coloration.

### The expression pattern of OvANS in different tissues of *O. violaceus*

**Fig. 6:**
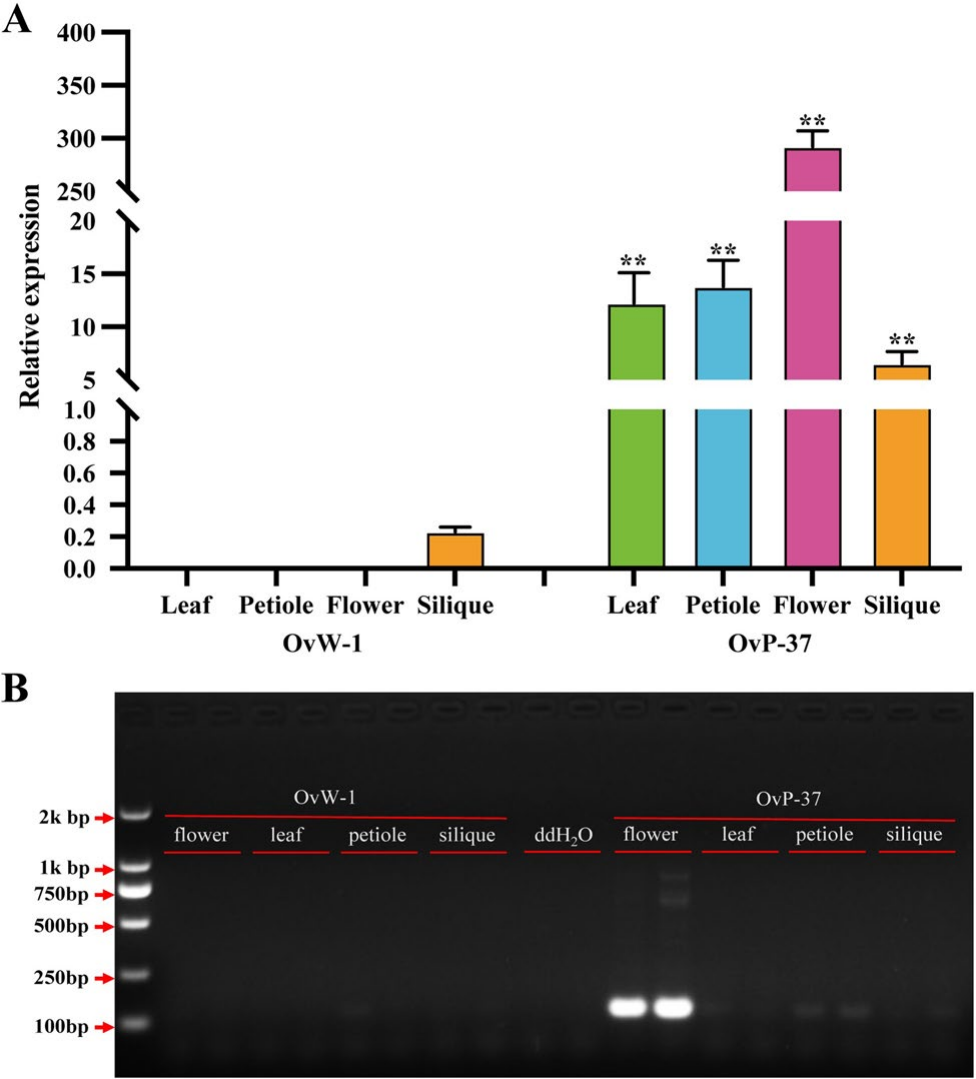
Expression pattern of *OvANS* in different tissues of *O. violaceus* OvP-37 and OvW-1 inbred lines. **A**: qRT-PCR analysis, *t*-test significant difference: ***, *P* < 0.001. Error bars, + SE from three biological repetitions; **B**: Semi-quantitative verification

To further investigate the expression pattern of *OvANS* in various tissues of *O. violaceus*, we conducted qRT-PCR verification on the leaves, petioles, petals, and young siliques of the OvP-37 and OvW-1 inbred lines. Our findings indicate that *OvANS* expression was only minimally detected in young siliques of OvW-1 inbred lines, whereas it was significantly up-regulated in OvP-37 inbred lines, particularly in petals (Fig. 6A). Subsequently, we utilized cDNA from leaves, petioles, petals, and young siliques to semi-quantitatively analyze the expression pattern of *OvANS.* The findings were in alignment with the results of qRT-PCR validation, indicating the presence of *OvANS* in the young siliques of OvW-1 inbred lines. While faint bands were observed in OvP-37 inbred lines, the most prominent bands were detected in petals (Fig. 6B). These results further demonstrated that sequence insertion mutation in the *OvANS* promoter region may be the main reason for the abnormal expression of *OvANS* in the OvW-1 inbred line. Conversely, *OvANS* was found to be expressed normally in OvP-37 inbred lines, particularly in petals.

## Discussion

*O. violaceus* is a species of significant phylogenetic importance in elucidating the evolutionary trajectory of the Brassicaceae family, characterized by a unique whole-genome duplication event specific to the *Orychophragmus* genus [24, 25]. With a rich history of cultivation and widespread popularity as an early-flowering ornamental plant in China, *O. violaceus* was the subject of a study employing comparative transcriptome sequencing, qRT-PCR validation, and gene cloning analysis. Our findings suggest that the *OvANS* gene may play a pivotal role in the suppression of anthocyanin biosynthesis in *O. violaceus*. Our RNA-seq analysis revealed that differential expression of the *OvANS* gene played a crucial role in determining the coloration of purple and white petals, a finding corroborated by qRT-PCR analysis. Upon cloning the full-length sequence of the *OvANS* gene from purple and white flowers, we identified two significant sequence insertions in the *OvANS* promoter region of white flowers, leading to diminished expression of the *OvANS* gene and virtually no expression of the *OvANS* gene in various tissues of *O. violaceus*, including leaves, petioles, petals, and pods. This mutation may be attributed to a mutation in either a late structural gene or a transcriptional regulatory factor that inhibits the modification of anthocyanin coloration.

### Fragment insertion in the promoter region represses *OvANS* in *O. violaceus*

The findings of this study indicate that the expression of the *OvANS* gene is absent in various plant tissues of white flower *O. violaceus* due to the presence of two inserted fragments in the promoter region. This suggests that the insertion of these fragments may impede the transcription of *OvANS*, thereby disrupting its normal functionality. Genetic events in plants, such as transposon mobilization, chromosomal rearrangements, and recombination, frequently lead to the insertion and deletion of gene promoter regions and fragments, affecting gene expression. Insertions or deletions in genetic sequences often result in alterations in gene expression levels, such as the activation or inhibition of gene expression. For instance, in purple-head Chinese cabbage (*B. rapa*), the presence of a short intron 1 of *BrMYB2* has been found to regulate anthocyanin biosynthesis [38]. Additionally, instances of gene expression activation resulting from fragment insertions have been documented, as seen in *B. oleracea* where different types of fragments inserted in the promoter region of the *BoMYB2* gene contribute to color variations among its subspecies [39]. Previous research has demonstrated that the insertion of bases in the promoter region of *BnaPAP2.A7* is crucial for inducing its expression in *B. napus* leaves, resulting in the production of three distinct transcripts with potential functional differences [34]. Similarly, in *B. napus* petals, the insertion of specific fragments in the promoter region of *BnaA07.PAP2* leads to heightened expression, which plays a pivotal role in anthocyanin biosynthesis and accumulation [40]. Furthermore, in the purple stems and orange petals of *B. napus*, differential expression is attributed to the insertion and deletion of specific regions within the *BnaPAP2.C6a* and *BnaPAP2.A7b* promoter regions or introns [41]. In a comparable manner, the 1,033-bp insertion in *B. juncea* has been shown to stimulate the expression of *BjMYB113c* in purple leaves [42], underscoring the significance of allelic sequence diversity in shaping plant genetic variability. For instance, the aberrant expression of the promoter region of the *OvANS* gene in *O. violaceus*, caused by a fragment insertion, results in a shift in flower color from purple to white.

### ANS plays a central role in anthocyanin biosynthesis in plant species

The biosynthetic pathway of plant anthocyanins is well-established, with key components including structural gene-encoded proteins that are catalytic in nature. The expression of these structural genes is primarily controlled by the MBW transcription complex [35]. Within plants, the *ANS* gene encodes leucoanthocyanidin dioxygenase, which plays a crucial role in anthocyanin biosynthesis and predominantly governs this process in vegetative organs and petals. Conversely, the functionally analogous gene anthocyanin reductase (ANR) primarily regulates anthocyanin biosynthesis in the seed coat [35, 43]. Our qRT-PCR analysis revealed that only *OvANS* exhibited low expression in siliques of OvW-1. This may be attributed to the primer specificity to the *ANR* gene, as evidenced by the brown seed coat color of OvW-1. Previous studies have shown that *ANS* is typically highly expressed in purple tissues or organs in Chinese cabbage [38], *B. oleracea* [39], *B. napus* [34, 40, 41], and *B. juncea* [42] due to the biological and cumulative color variation of anthocyanins. In *B. juncea*, the upregulation of *ANS* genes (*BjuA004031*, *BjuB014115*, *BjuB044852*, *BjuO009605*) is crucial for the development of purple veins [44]. Our prior investigation on *B. rapa* also revealed that the insertion of two small fragments in the *BraANS.A3* promoter region led to diminished expression, resulting in green foliage [33]. These findings underscore the pivotal role of *ANS* in the biosynthesis of plant anthocyanins, and indicate that the suppression of *ANS* gene activity can impede the anthocyanin biosynthesis pathway in plants, manifesting in a phenotype devoid of anthocyanin synthesis and accumulation. However, the function of *OvANS* remains to be further verified.

## Conclusion

This study initially focused on identifying the genes associated with the anthocyanin biosynthetic pathway of *O. violaceus* at the whole genome level. Subsequently, a comparative transcriptome sequencing approach was employed to investigate the differential expression of genes related to anthocyanin biosynthesis in the petals of the white flower mutant and the purple wild-type flower. This analysis was further validated using qRT-PCR. The findings suggest that the notably differential expression of *OvANS* may play a pivotal role in determining the color variation between purple and white flowers. Additionally, the full-length sequence of the *OvANS* gene was cloned from both the white-flowered mutant and purple-flowered wild-type inbred line of *O. violaceus*. Analysis revealed that the insertion of two fragments in the promoter region may play a crucial role in suppressing the expression of the *OvANS* gene. Subsequently, qRT-PCR was utilized to assess the expression of the *OvANS* gene in the leaves, petioles, petals, and siliques of *O. violaceus*. It was observed that *OvANS* and *OvPAP1* were minimally expressed in the four tissues of the white flower mutant, whereas *OvANS* exhibited normal expression in the purple flower wild type. The findings of this study demonstrate that the insertion of the *OvANS* gene promoter fragment in the white flower mutant of *O. violaceus* leads to the repression of gene expression, resulting in the inhibition of anthocyanin biosynthesis and subsequent anthocyanin deficiency throughout the plant. These results contribute to the understanding of the molecular mechanisms underlying flower color variation in *O. violaceus* and offer insights for enhancing the diversity of flower colors in this species.

## Supplementary Information

Below is the link to the electronic supplementary material.

Supplementary Material 1.

Supplementary Material 2.

Supplementary Material 3.

Supplementary Material 4.

Supplementary Material 5.

## Data Availability

The RNA-seq data used in this study were generated by the authors and uploaded to NCBI (https://www.ncbi.nlm.nih.gov/) with biological projects PRJNA1094639.

## References

[1] Koes R, Verweij W, Quattrocchio F. Flavonoids: a colorful model for the regulation and evolution of biochemical pathways. Trends Plant Sci. 2005;10(5):236–42. 15882656 10.1016/j.tplants.2005.03.002

[2] Stuurman J, Hoballah ME, Broger L, Moore J, Basten C, Kuhlemeier C. Dissection of floral pollination syndromes in Petunia. Genetics. 2004;168(3):1585–99. 15579709 10.1534/genetics.104.031138 PMC1448759

[3] Zafra-Stone S, Yasmin T, Bagchi M, Chatterjee A, Vinson JA, Bagchi D. Berry anthocyanins as novel antioxidants in human health and disease prevention. Mol Nutr Food Res. 2007;51(6):675–83. 17533652 10.1002/mnfr.200700002

[4] Zhang Y, Butelli E, Martin C. Engineering anthocyanin biosynthesis in plants. Curr Opin Plant Biol. 2014;19:81–90. 24907528 10.1016/j.pbi.2014.05.011

[5] Bendokas V, Skemiene K, Trumbeckaite S, Stanys V, Passamonti S, Borutaite V, et al. Anthocyanins: from plant pigments to health benefits at mitochondrial level. Crit Rev Food Sci Nutr. 2020;60(19):3352–65. 31718251 10.1080/10408398.2019.1687421

[6] Shi MZ, Xie DY. Biosynthesis and metabolic engineering of anthocyanins in *Arabidopsis thaliana*. Recent Pat Biotechnol. 2014;8(1):47–60. 24354533 10.2174/1872208307666131218123538 PMC4036305

[7] Tohge T, Nishiyama Y, Hirai MY, Yano M, Nakajima J, Awazuhara M, et al. Functional genomics by integrated analysis of metabolome and transcriptome of *Arabidopsis* plants over-expressing an MYB transcription factor. Plant J. 2005;42(2):218–35. 15807784 10.1111/j.1365-313X.2005.02371.x

[8] Rowan DD, Cao M, Lin-Wang K, Cooney JM, Jensen DJ, Austin PT, et al. Environmental regulation of leaf colour in red 35S:PAP1 *Arabidopsis thaliana*. New Phytol. 2009;182(1):102–15. 19192188 10.1111/j.1469-8137.2008.02737.x

[9] Stracke R, Jahns O, Keck M, Tohge T, Niehaus K, Fernie AR, et al. Analysis of production of flavonol glycosides-dependent flavonol glycoside accumulation in *Arabidopsis thaliana* plants reveals MYB11-, MYB12- and MYB111-independent flavonol glycoside accumulation. New Phytol. 2010;188(4):985–1000. 20731781 10.1111/j.1469-8137.2010.03421.x

[10] Petroni K, Tonelli C. Recent advances on the regulation of anthocyanin synthesis in reproductive organs. Plant Sci. 2011;181(3):219–29. 21763532 10.1016/j.plantsci.2011.05.009

[11] Jin H, Cominelli E, Bailey P, Parr A, Mehrtens F, Jones J, Tonelli C, Weisshaar B, Martin C. Transcriptional repression by AtMYB4 controls production of UV-protecting sunscreens in Arabidopsis. Embo J. 2000;19(22):6150–61. 11080161 10.1093/emboj/19.22.6150 PMC305818

[12] Zimmermann IM, Heim MA, Weisshaar B, Uhrig JF. Comprehensive identification of *Arabidopsis thaliana* MYB transcription factors interacting with R/B-like BHLH proteins. Plant J. 2004;40(1):22–34. 15361138 10.1111/j.1365-313X.2004.02183.x

[13] Stracke R, Ishihara H, Huep G, Barsch A, Mehrtens F, Niehaus K, et al. Differential regulation of closely related R2R3-MYB transcription factors controls flavonol accumulation in different parts of the *Arabidopsis thaliana* seedling. Plant J. 2007;50(4):660–77. 17419845 10.1111/j.1365-313X.2007.03078.x PMC1976380

[14] Jaakola L. New insights into the regulation of anthocyanin biosynthesis in fruits. Trends Plant Sci. 2013;18(9):477–83. 23870661 10.1016/j.tplants.2013.06.003

[15] Zhao L, Gao L, Wang H, Chen X, Wang Y, Yang H, Wei C, Wan X, Xia T. The R2R3-MYB, bHLH, WD40, and related transcription factors in flavonoid biosynthesis. Funct Integr Genomics. 2013;13(1):75–98. 23184474 10.1007/s10142-012-0301-4

[16] Dubos C, Stracke R, Grotewold E, Weisshaar B, Martin C, Lepiniec L. MYB transcription factors in Arabidopsis. Trends Plant Sci. 2010;15(10):573–81. 20674465 10.1016/j.tplants.2010.06.005

[17] LaFountain AM, Yuan YW. Repressors of anthocyanin biosynthesis. New Phytol. 2021;231(3):933–49. 33864686 10.1111/nph.17397 PMC8764531

[18] Nesi N, Debeaujon I, Jond C, Pelletier G, Caboche M, Lepiniec L. The *TT8* gene encodes a basic helix-loop-helix domain protein required for expression of *DFR* and *BAN* genes in Arabidopsis siliques. Plant Cell. 2000;12(10):1863–78. 11041882 10.1105/tpc.12.10.1863 PMC149125

[19] Gonzalez A, Zhao M, Leavitt JM, Lloyd AM. Regulation of the anthocyanin biosynthetic pathway by the TTG1/bHLH/Myb transcriptional complex in Arabidopsis seedlings. Plant J. 2008;53(5):814–27. 18036197 10.1111/j.1365-313X.2007.03373.x

[20] Li X, Teitgen AM, Shirani A, Ling J, Busta L, Cahoon RE, et al. Discontinuous fatty acid elongation yields hydroxylated seed oil with improved function. Nat Plants. 2018;4(9):711–20. 30150614 10.1038/s41477-018-0225-7

[21] Li ZY, Ge XH. Unique chromosome behavior and genetic control in brassica x *Orychophragmus* wide hybrids: a review. Plant Cell Rep. 2007;26(6):701–10. 17221227 10.1007/s00299-006-0290-7

[22] Xu C, Huang Q, Ge X, Li Z. Phenotypic, cytogenetic, and molecular marker analysis of *brassica napus* introgressants derived from an intergeneric hybridization with *orychophragmus*. PLoS ONE. 2019;14(1):e0210518. 30629679 10.1371/journal.pone.0210518 PMC6328085

[23] Fu W, Chen D, Pan Q, Li F, Zhao Z, Ge X, et al. Production of red-flowered oilseed rape via the ectopic expression of *Orychophragmus violaceus* OvPAP2. Plant Biotechnol J. 2018;16(2):367–80. 28640973 10.1111/pbi.12777 PMC5787836

[24] Huang F, Chen P, Tang XY, Zhong T, Yang TH, Nwafor CC, et al. Genome assembly of the brassicaceae diploid *Orychophragmus violaceus* reveals complex whole-genome duplication and evolution of dihydroxy fatty acid metabolism. Plant Commun. 2023. 10.1016/j.xplc.2022.100432. 36071666 10.1016/j.xplc.2022.100432 PMC10030321

[25] Zhang K, Yang Y, Zhang X, Zhang L, Fu Y, Guo Z, et al. The genome of *Orychophragmus violaceus* provides genomic insights into the evolution of brassicaceae polyploidization and its distinct traits. Plant Commun. 2023;4(2):100431. 36071668 10.1016/j.xplc.2022.100431 PMC10030322

[26] Chen DZ, Yang YX, Niu GB, Shan XZ, Zhang XL, Jiang HM, et al. Metabolic and RNA sequencing analysis of cauliflower curds with different types of pigmentation. AOB Plants. 2022. 10.1093/aobpla/plac001. 35414860 10.1093/aobpla/plac001 PMC8994856

[27] Bolger AM, Lohse M, Usadel B. Trimmomatic: a flexible trimmer for illumina sequence data. Bioinformatics. 2014;30(15):2114–20. 24695404 10.1093/bioinformatics/btu170 PMC4103590

[28] Pertea M, Kim D, Pertea GM, Leek JT, Salzberg SL. Transcript-level expression analysis of RNA-seq experiments with HISAT, stringtie and ballgown. Nat Protoc. 2016;11(9):1650–67. 27560171 10.1038/nprot.2016.095 PMC5032908

[29] Chen CJ, Wu Y, Li JW, Wang X, Zeng ZH, Xu J, et al. TBtools-II: a one for all, all for onebioinformatics platform for biological big-data mining. Mol Plant. 2023;16(11):1733–42. 37740491 10.1016/j.molp.2023.09.010

[30] Love MI, Huber W, Anders S. Moderated estimation of fold change and dispersion for RNA-seq data with DESeq2. Genome Biol. 2014;15(12):550. 25516281 10.1186/s13059-014-0550-8 PMC4302049

[31] Wang Y, Tang H, Debarry JD, Tan X, Li J, Wang X, et al. MCscanx: a toolkit for detection and evolutionary analysis of gene synteny and collinearity. Nucleic Acids Res. 2012;40(7):e49. 22217600 10.1093/nar/gkr1293 PMC3326336

[32] Kumar S, Stecher G, Tamura K. MEGA7: molecular evolutionary genetics analysis version 7.0 for bigger datasets. Mol Biol Evol. 2016;33(7):1870–4. 27004904 10.1093/molbev/msw054 PMC8210823

[33] Tan C, Chen H, Dai G, Liu Y, Shen W, Wang C, et al. Identification and characterization of the gene BraANS.A03 associated with purple leaf color in Pak Choi (*Brassica rapa* L. ssp. chinensis). Planta. 2023;258(1):19. 37314587 10.1007/s00425-023-04171-7

[34] Chen D, Liu Y, Yin S, Qiu J, Jin Q, King GJ, Wang J, Ge X, Li Z. Alternatively spliced BnaPAP2.A7 isoforms play opposing roles in anthocyanin biosynthesis of brassica Napus L. Front Plant Sci. 2020;11:983. 32973819 10.3389/fpls.2020.00983 PMC7466728

[35] Xu W, Dubos C, Lepiniec L. Transcriptional control of flavonoid biosynthesis by MYB-bHLH-WDR complexes. Trends Plant Sci. 2015;20(3):176–85. 25577424 10.1016/j.tplants.2014.12.001

[36] Schuurink RC, Haring MA, Clark DG. Regulation of volatile benzenoid biosynthesis in Petunia flowers. Trends Plant Sci. 2006;11(1):20–5. 16226052 10.1016/j.tplants.2005.09.009

[37] Albert NW, Butelli E, Moss SMA, Piazza P, Waite CN, Schwinn KE, Davies KM, Martin C. Discrete bHLH transcription factors play functionally overlapping roles in pigmentation patterning in flowers of. New Phytol. 2021;231(2):849–63. 33616943 10.1111/nph.17142 PMC8248400

[38] He Q, Wu J, Xue Y, Zhao W, Li R, Zhang L. The novel gene BrMYB2, located on chromosome A07, with a short intron 1 controls the purple-head trait of Chinese cabbage (Brassica Rapa L). Hortic Res. 2020;7:97. 32637125 10.1038/s41438-020-0319-z PMC7326913

[39] Yan C, An G, Zhu T, Zhang W, Zhang L, Peng L, et al. Independent activation of the BoMYB2 gene leading to purple traits in *brassica oleracea*. Theor Appl Genet. 2019;132(4):895–906. 30467611 10.1007/s00122-018-3245-9

[40] Ye SH, Hua SJ, Ma TT, Ma XW, Chen YP, Wu LM, Zhao L, Yi B, Ma CZ, Tu JX, et al. Genetic and multi-omics analyses reveal BnaA07.PAP2 as the key gene conferring anthocyanin-based color in brassica Napus flowers. J Exp Bot. 2022;73(19):6630–45. 35857343 10.1093/jxb/erac312

[41] Chen D, Jin Q, Pan J, Liu Y, Tang Y, E Y, et al. Fine mapping of genes controlling pigment accumulation in oilseed rape (*Brassica napus* L). Mol Breed. 2023;43(3):19. 37313299 10.1007/s11032-023-01365-5 PMC10248657

[42] An G, Chen J. Frequent gain- and loss-of-function mutations of the BjMYB113 gene accounted for leaf color variation in Brassica juncea. BMC Plant Biol. 2021;21(1):301. 10.1186/s12870-021-03084-5 PMC8240407 34187365

[43] Xu WJ, Grain D, Bobet S, Le Gourrierec J, Thévenin J, Kelemen Z, Lepiniec L, Dubos C. Complexity and robustness of the flavonoid transcriptional regulatory network revealed by comprehensive analyses of MYB-bHLH-WDR complexes and their targets in Arabidopsis seed. New Phytol. 2014;202(1):132–44. 24299194 10.1111/nph.12620

[44] Zhang K, Yang D, Hu Y, Njogu MK, Qian J, Jia L, Yan C, Li Z, Wang X, Wang L. Integrated analysis of transcriptome and metabolome reveals new insights into the formation of purple leaf veins and leaf edge cracks in brassica juncea. Plants. 2022;11(17):2229. 36079611 10.3390/plants11172229 PMC9460116

