# Supplementary figures and images for: Identification and characterization of the gene OvANS associated with purple flower in Orychophragmus violaceus
Source: BMC Plant Biol. 2025 Oct 9;25:1349. doi: 10.1186/s12870-025-07412-x (PMC12512534; doi:10.1186/s12870-025-07412-x)

**The original versions of Figure 6B.**


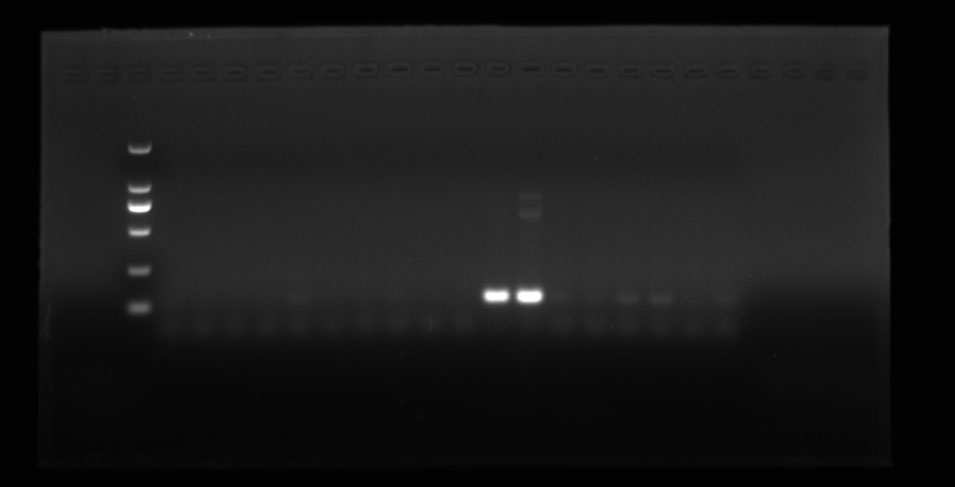


**The Figure 6B with documents.**


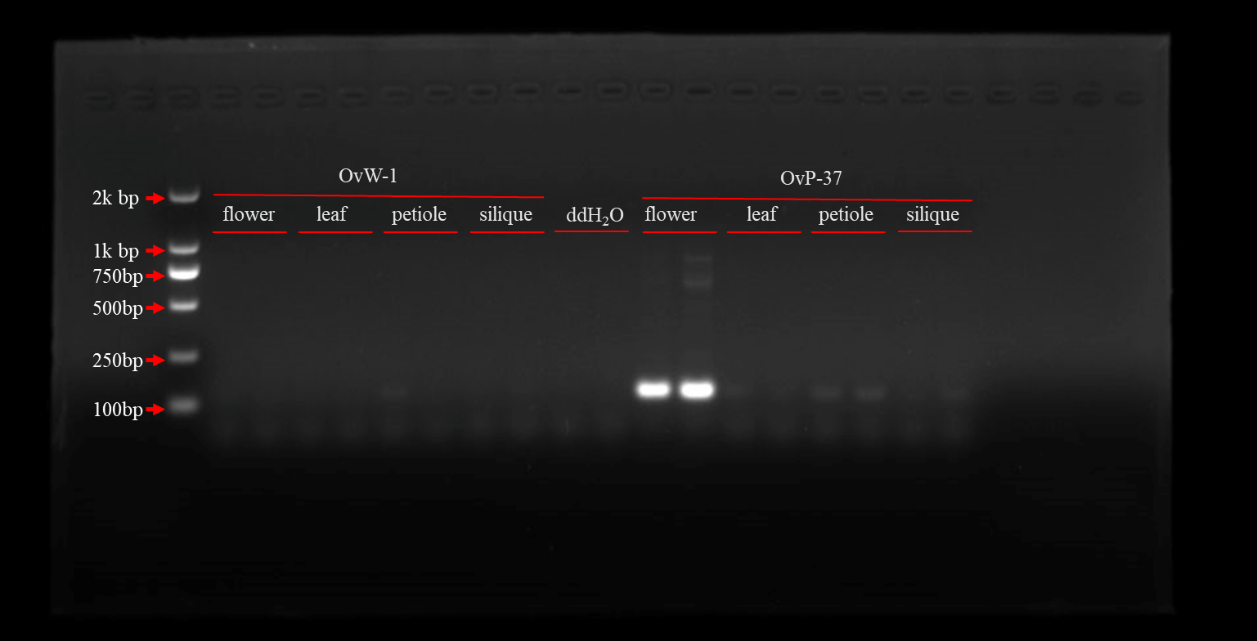

Supplement: Supplementary file 5 — Supplementary Material 5. [file 12870_2025_7412_MOESM5_ESM.docx]
